# Standardization of Preclinical PET/CT Imaging to Improve Quantitative Accuracy, Precision, and Reproducibility: A Multicenter Study

**DOI:** 10.2967/jnumed.119.231308

**Published:** 2020-03

**Authors:** Wendy McDougald, Christian Vanhove, Adrienne Lehnert, Barbara Lewellen, John Wright, Marco Mingarelli, Carlos Alcaide Corral, Jurgen E. Schneider, Sven Plein, David E. Newby, Andy Welch, Robert Miyaoka, Stefaan Vandenberghe, Adriana Alexandre S. Tavares

**Affiliations:** 1 BHF-Centre for Cardiovascular Science, College of Medicine & Veterinary Medicine, Queen’s Medical Research Institute, University of Edinburgh, United Kingdom; 2 Edinburgh Preclinical Imaging (EPI), Edinburgh Imaging, University of Edinburgh, Edinburgh, United Kingdom; 3 Department of Electronics and Information Systems, MEDISIP, Ghent University, Ghent, Belgium; 4 Department of Radiology, Imaging Research Laboratory, University of Washington, Seattle, Washington; 5 Leeds Institute of Cardiovascular and Metabolic Medicine, Department of Biomedical Imaging Science, LIGHT Laboratories, University of Leeds, Leeds, United Kingdom; and; 6 Aberdeen Biomedical Imaging Centre, School of Medicine, Medical Sciences & Nutrition, University of Aberdeen, Aberdeen, United Kingdom

**Keywords:** preclinical PET/CT, standardization, Hounsfield units, absorbed dose, recovery coefficient

## Abstract

Preclinical PET/CT is a well-established noninvasive imaging tool for studying disease development/progression and the development of novel radiotracers and pharmaceuticals for clinical applications. Despite this pivotal role, standardization of preclinical PET/CT protocols, including CT absorbed dose guidelines, is essentially nonexistent. This study (1) quantitatively assesses the variability of current preclinical PET/CT acquisition and reconstruction protocols routinely used across multiple centers and scanners; and (2) proposes acquisition and reconstruction PET/CT protocols for standardization of multicenter data, optimized for routine scanning in the preclinical PET/CT laboratory. **Methods:** Five different commercial preclinical PET/CT scanners in Europe and the United States were enrolled. Seven different PET/CT phantoms were used for evaluating biases on default/general scanner protocols, followed by developing standardized protocols. PET, CT, and absorbed dose biases were assessed. **Results:** Site default CT protocols were the following: greatest extracted Hounsfield units (HU) were 133 HU for water and −967 HU for air; significant differences in all tissue equivalent material (TEM) groups were measured. The average CT absorbed doses for mouse and rat were 72 mGy and 40 mGy, respectively. Standardized CT protocol were the following: greatest extracted HU were −77 HU for water and −990 HU for air; TEM precision improved with a reduction in variability for each tissue group. The average CT absorbed dose for mouse and rat decreased to 37 mGy and 24 mGy, respectively. Site default PET protocols were the following: uniformity was substandard in one scanner, recovery coefficients (RCs) were either over- or underestimated (maximum of 43%), standard uptake values (SUVs) were biased by a maximum of 44%. Standardized PET protocols were the following: scanner with substandard uniformity improved by 36%, RC variability decreased by 13% points, and SUV accuracy improved to 10%. **Conclusion:** Data revealed important quantitative biases in preclinical PET/CT and absorbed doses with default protocols. Standardized protocols showed improvements in measured PET/CT accuracy and precision with reduced CT absorbed dose across sites. Adhering to standardized protocols generates reproducible and consistent preclinical imaging datasets, thus augmenting translation of research findings to the clinic.

The necessity for standardization in clinical PET/CT protocols was acknowledged and initiated nearly 20 y ago (1–4). This recognition mainly stemmed from multicenter trials focused on quantifying and tracking changes in malignant tumors as well as prognosis and treatment evaluations. Today preclinical PET/CT is a pivotal quantitative imaging research tool supporting innovative research in areas such as disease diagnosis and prognosis and in the development of novel radiotracers and pharmaceuticals (5–10). Yet, standardization in preclinical PET/CT imaging remains essentially nonexistent. To date there is not an established global standard protocol used across preclinical research centers. The lack of preclinical protocol standardization impacts quantitative image analysis, reproducibility, and consistency across sites, thus limiting reliable translational image data to clinical research and applications.

The preclinical PET/CT community has undertaken efforts toward the development of guidelines regarding animal handling/preparation and scanner quality control testing (11–17). Several preclinical studies evaluating PET National Electric Manufactures Association (NEMA) NU 4 2008 performance also exist (NEMA performance literature in Supplemental Table 1; supplemental materials are available at http://jnm.snmjournals.org) (1–13). However, not until the present study has establishing preclinical imaging standard protocols been directly addressed and set forth. Additionally, due to unregulated preclinical CT doses absorbed ionized radiation is assessed, fostering the impetus for regulating ionizing absorbed radiation doses (18). Regulating CT doses will reduce the cumulative severity effects of radiation. This will minimize animal suffering while reducing the potential impact of biologic responses from the radiation effect on research studies, in line with the National Centre for the Replacement, Refinement and Reduction of Animals in Research (NC3Rs).

This study addresses the lack of standardized protocols by assessing quantitative accuracy (known vs. measured) and precision (reduced variability) of currently used routine protocols across multiple sites and scanners for the development of standard protocols.

## MATERIALS AND METHODS

This multicenter study involved 5 sites for a total of 5 different commercial preclinical PET/CT scanners (Bruker Albira, Mediso nanoPET/CT, Sedecal Super Argus, Siemens Inveon and Trifoil LabPET/CT), arbitrarily labeled 1 to 5. First, routine/default (hereon referred to as default) PET and CT protocols were evaluated for image quality and quantification biases using seven commercially available preclinical microPET and microCT phantoms (Supplemental Table 2, labeled A–G). Default protocols were set either by the vendor or by the site for their routine use of imaging small animals. Each CT default protocol was also assessed based on measured absorbed ionizing radiation. Second, several different PET reconstruction methods were quantitatively analyzed for standardization. Third, standardized CT protocols were determined from the least Hounsfield units (HU) biases between all imaging data sets. Numerical criteria for biases were based on the parameters in Table 1. Results were then evaluated in the same manner as the default protocols on each scanner. All PET and CT imaging data sets (default and standard) per scanner per phantom and dose measurements were acquired as *n* = 3 for the analysis. No rodents were used in this study, only dedicated PET and CT phantoms. The lead author visited each site multiple times for the image acquisitions and carried out all the data analysis.

**TABLE 1:** Criteria for Quantitative Analysis of PET and CT Results

Parameter	Data
PET IQ phantom	
Uniformity	<15%
RCs	1
SOR	<0.20
SUV	<10% bias
CT air/water phantom	
Air	0 HU
Water	−1,000 HU
CT TEM phantom	
Lung	−700 HU
Soft tissue (adipose/muscle)	>0 HU
Bone (soft/cortical)	>200 HU

*HU for TEM originally defined based on literature (27).

### Default PET/CT Protocols

#### PET

PET images were acquired as a single bed position for a duration of 20-min, energy windowing of 250–700 keV with the phantoms placed at the front end of the scanner bed, positioned inside the bore at the isocenter, aligning sagittal, axial, and coronal planes. An activity of 10 ± 6 MBq of ^18^F-FDG in 23 mL of distilled water was injected into a PET image quality (IQ) phantom, which includes 5 hots rods 1–5 mm for recovery coefficient (RC), uniformity section, and spillover-ratio (SOR) section composed of 2 cylinders filled with nonradioactive water and air. ^18^F-FDG (64 ± 5 MBq) in 24 mL of distilled water was injected into a PET rod phantom containing 0.6, 0.8, 1.0, 1.2, 1.5 and 2.0 mm rods. The targeted range of activities was selected based on typically reported injected doses into small animals, the design and purpose of the PET phantoms.

Emission data were reconstructed using the sites' default protocols. Protocols are listed by scanner (1–5), method (ordered-subset expectation maximization [OSEM], or maximum likelihood expectation maximization [MLEM]), voxel size, filter, and matrix size: 1 (2D OSEM 2 iterations 16 subsets, 0.4 mm, Ramp, 175 × 175), 2 (3D OSEM 4 iterations 6 subsets, 0.4 mm, Ramp, 108 × 110), 3 (3D OSEM 2 iterations 18 subsets, 0.3 mm, Hamm, 256 × 256), 4 (3D MLEM 12 iterations, 0.7 mm, no filter,108 × 108), and 5 (2D MLEM 50 iterations, 0.5 mm, no filter, 200 × 200). Scanners 2 and 3 also correct for partial-volume effects by incorporating a point spread function (PSF) into the reconstruction algorithm. All scanners apply scatter, normalization and randoms corrections, whereas scanners 1–4 apply attenuation corrections. Scanner 5 allows the user to opt out of using the CT-generated attenuation correction maps.

For the PET IQ image analysis, reconstructed data were imported into PMOD version 3.806 (PMOD) and a MATLAB software tool implemented by Mediso. The Mediso MATLAB software program utilizes the NEMA NU 4-2008 standards. The quantitative assessment of the PET included uniformity, RC, SOR and standard uptake values (SUV).

#### PET Statistical Analysis

In accordance to NEMA, using the IQ phantom, uniformity is reported as the percent standard deviation (%STD) from a 22.5 mm diameter by 10 mm long cylindrical volume of interest (VOI) over the uniform region of the phantom. RC is calculated based on values extracted from regions of interest (ROI) twice the diameter of each hot rod. The MATLAB program draws linear profiles along the hot rods in the axial direction. The mean pixel values of the linear profiles are divided by the mean pixel value of the uniform region, Equation 1 below (19,20).

Eq. 1 $$RC=\frac{ROI_{rod}}{VOI_{uniformity}}.$$

*ROI*_*rod*_ represents the mean pixel values from the hot rods (1, 2, 3, 4 and 5 mm), and *VOI*_*uniformity*_ is the mean activity concentration from the uniformity region.

VOIs are drawn on each air and water chamber with SOR values calculated as ratios between the air or water chamber mean value divided by the uniformity mean measurement, Equation 2 (19,20).

Eq. 2 $$SOR=\frac{VOI_{chamber}}{VOI_{uniformity}}.$$

*VOI*_*chamber*_ represents the mean value from each individual air or water chamber and *VOI*_*uniformity*_ is the uniformity measurement. Representative images of the Mediso MATLAB software tool for the PET IQ analysis displaying the regions of the IQ phantom (uniformity, RC, and SOR) as well as the placements of the drawn regions/volumes of interest are shown in Supplemental Figure 1A.

SUV results were obtained first using PMOD’s SUV image calculation scaler tool with a phantom measured weight of 0.073 kg. After scaling, a 2.8 mL VOI template was placed on the uniformity section of the PET IQ phantom for the extraction of SUV results. A representative image of VOI placement on the PET IQ phantom is shown in Supplemental Figure 1B. For analysis of variance, an ordinary 1-way (ANOVA) test was applied on the SUV data: default, standard and filter back projection (FBP).

PET spatial resolution assessment was conducted based on a visual assessment of the acquired images using the PET rod phantom. Horizontal profiles (H-profile) were drawn through a center cross section, which included the largest rods (2 mm) obtained using the PMOD image profile tool.

#### CT

The phantoms were placed at the front end of the scanner bed, positioned inside the bore at the isocenter, aligning sagittal, axial, and coronal planes. For each default protocol, CT basic acquisition parameters varied by tube voltages (kVp), number of projections, and exposure time (ms) by scanner as follows: 1 (40 kVp, 360, 300 ms), 2 (50 kVp, 480, 300 ms), 3 (80 kVp, 220, 280 ms), 4 (35 kVp, 250, 300 ms), and 5 (50 kVp, 256, 555 ms). CT protocol parameters per scanner are listed in Supplemental Table 3. All CT images were reconstructed with FBP.

#### CT Statistical Analysis

Reconstructed CT data were imported into PMOD for analysis. A 5 mL VOI was placed on the air and water chamber of the CT air/water phantom to quantify the mean HU values (Supplemental Fig. 2A). The TEM phantom data were imported into PMOD and individually coregistered with an in-house–developed TEM phantom template (Supplemental Fig. 2B), in order to ensure correct and consistent placement of VOIs on each rod for each CT image. A VOI template was generated for each rod (0.008 mL for 2 mm and 0.05 mL for 4 mm) for extraction of HUs (Supplemental Fig. 2B). HU quantification accuracy and precision was defined as bias between measured HU relative to established HU value for air, water, and tissue (Table 1). The data are represented as the mean ± standard deviation (SD). Precision is assessed by measuring the SD and coefficient of variation (COV). For analysis of variance, an ordinary 1-way (ANOVA) test was applied on the TEM data, grouped per tissue density.

CT image spatial resolution was evaluated by visual assessment of the image obtained with the spatial resolution bar phantom. The number of structures (lines/dots with widths varying between 5 and 150 µm) on the bar pattern seen were compared to the manufacturer’s size chart to estimate each protocol spatial resolution.

### Measurement of Ionizing Radiation Doses from CT Acquisition Protocols

An ionization chamber probe (10x6-0.6 CT Therapy QA Chamber, detection range 1 μGy–5 kGy with ±4% calibration accuracy; Radcal) was used for radiation dose measurements. The ion chamber probe was placed inside the CT dose index (CTDI) phantoms with the chamber in the center field of view (FOV). Default and standardized CT protocol measurements were obtained (*n* = 3) on all scanners with the mouse and rat CTDI phantom.

The Radcal ion chamber software stops collecting/measuring at 300 s. We previously showed the measured CT dose with the RadCal probe is linearly dependent on scan length (21). Therefore, CT protocols with a scan time longer than 5 min were measured to 300 s then the dose was calculated based on remaining frames and measured dose (Eq. 3).

Eq. 3 $$\mathrm{Measured}\;\mathrm{CT}\;\mathrm{dose}=\left(\frac{Frames_{protocol}}{Frames_{acquired}}\right)\;Dose_{measured}.$$

### Standardized PET/CT Protocols

Developed standardized protocols were derived from the default protocol analysis results as described above and in Table 1. The actual PET and CT parameters available on each manufacturer’s scanner were also taken into consideration.

Standardizing the PET protocol entailed evaluating the impact different reconstruction methods had on the quantification of the PET image data sets. The following reconstruction algorithms were tested: FBP, OSEM with a combination of iterations*subsets of 12, 16, 24, 30, 32, 48, and 64, and MLEM with 12, 24, 25, 30, 32, 40, and 50 iterations. Quantitative analysis of the OSEM updates used in scanners 1, 2, and 3 revealed the optimal reconstruction methods were already being used for these particular scanners (Supplemental Table 4). For that reason, focus was placed on optimizing the MLEM method not only for improved accuracy but also for the best equivalent results to the OSEM method. Similarly, the MLEM method with 25 iterations provided lower quantitative bias compared with OSEM outcomes from scanners 1–3. The FBP algorithm was deemed to be the optimal method for least quantitative bias across all scanners. Consequently, in the results section, we report data for scanners’ 1–3 default OSEM methods, scanner 4 and 5 default and standardized MLEM with 25 updates, as well as FBP results for scanners 1–3 and 5.

From the analysis of the empirical CT data, 4 standardized CT protocols were developed and tested. The tube voltage was set at 50 kVp and exposure time at 300 ms for all scanners with 4 varying numbers of projections (170, 360, 480, and 720 projections). Not all the scanners could set the projection parameters at 170 or 480. In those cases, data were only collected for the remaining protocols. CT collected data, including CT absorbed doses, were analyzed in the same manner as the default protocols, as outlined above. In the results section, CT imaging data derived from default and standard acquisition protocols using 360 projections are presented, given that all scanners allowed for this setting.

## RESULTS

### Analysis of PET Acquisitions Using Default and Standardized Protocols

#### PET IQ

**FIGURE 1.**
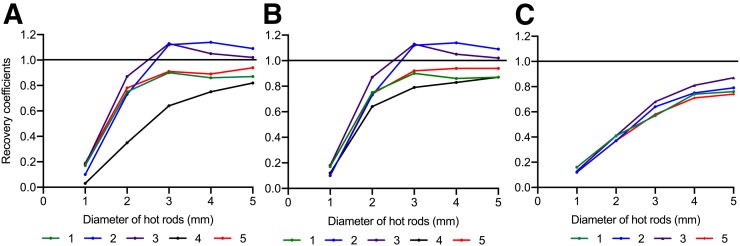
Recovery coefficients (RCs) for hot rods of 1, 2, 3, 4, and 5 mm of the PET IQ phantom extracted for each scanner. (A) Default reconstruction methods for scanners 1 (2D OSEM 2 iterations 16 subsets), 2 (3D OSEM 4 iterations 6 subsets, PSF), 3 (3D OSEM 2 iterations 18 subsets, PSF), 4 (3D MLEM 12 iterations), and 5 (2D MLEM 50 iterations). (B) RCs with standardization for scanner 4 (3D MLEM 25) and scanner 5 (2D MLEM 25) leaving scanners 1, 2, and 3 with the default reconstruction method. (C) RCs for each site using FBP reconstruction with the exception of scanner 4 (reconstruction option not available yet).

As seen in Fig. 1A, scanners 2 and 3 default reconstruction method overestimated the RCs by as much as 13% relative to 1 at the hot rod 3. Whereas, scanners 1, 4, and 5 default reconstruction method underestimated the RCs. The RCs measured for scanners 4 and 5 improved after implementing a standardized number of MLEM iterations at 25, shown in Fig. 1B. A 43% difference measured between scanner 3 and 4 at the 3 mm hot rod using default protocols was reduced to a 30% relative difference when using the standardized protocol. The FBP method produced the most consistent RCs of all methods (Fig. 1C).

Table 2

**TABLE 2:** PET IQ, Measured Uniformity, and Spill-Over Ratios (SOR) Using the Default Reconstruction Methods and the Standardized Reconstruction Method

	Default protocol			Standardized protocol		
Scanner	Uniformity (SD%)	SOR water	SOR air	Uniformity (SD%)	SOR water	SOR air
1	6.4±0.01	0.18±0.04	0.13±0.03	6.4±0.01	0.18±0.04	0.13±0.03
2	4.1±1.00	0.09±0.01	0.09±0.01	4.1±1.00	0.09±0.01	0.09±0.01
3	3.4±0.17	0.01±0.01	0.02±0.01	3.4±0.17	0.01±0.01	0.02±0.01
4	5.2±0.60	0.28±0.04	0.22±0.04	6.4±0.00	0.21±0.00	0.13±0.00
5	16.7±0.55	0.24±0.01	0.12±0.02	10.6±0.00	0.27±0.00	0.17±0.00

Values expressed as mean ± standard deviation, *n* = 3.

reveals poor image uniformity in scanner 5 before standardization. The standardized reconstruction protocol (MLEM 25) improved uniformity in scanner 5 by a relative percentage difference of 36% (i.e., 16.7% to 10.6%). Though protocol standardization improved scanner 5’s uniformity, there was no improvement in water and air SORs. This uniformity improvement was not observed in scanner 4 (MLEM 25), although its uniformity was already similar to OSEM data collected with other scanners. An improvement was seen in scanner 4's SORs for water and air. The mean uniformity value reduced by 12% when standardization was applied (improved COV from 67% to 37%).

Analysis of the SUV variance proved significant for the default protocols and nonsignificant for the standard and FBP protocols (ANOVA: Default *P* < 0.001, Standard *P* < 0.205, FBP *P* < 0.388 [FBP scanner 4 not included], *n* = 3 per group). The greatest percentage difference (44%) in SUVs obtained using default protocols was between scanner 2 and 4. This was reduced to 14% with standardization. Using FBP, the greatest percentage difference of 6% was measured between scanner 2 and 5. The percentage difference between the average expected SUV and the average default SUV or to the standardized SUV was 18% and 10%, respectively (Table 3 and Supplemental Fig. 3).

**TABLE 3:** Measured and Expected Standard Uptake Values (SUVs) for Each Scanner Using the Default, Standardized Reconstruction Iterative Method, and FBP

Scanner	Expected	Default	Measured/mean	Standard	Measured/mean	FBP	Measured/mean
Scanner 1	3.61±0.59	3.24±0.34	1.04±0.11	3.24±0.34	0.96±0.09	3.26±0.14	1.03±0.04
Scanner 2	3.87±0.62	3.77±1.06	1.21±0.34	3.77±1.06	1.12±0.31	3.18±0.39	1.00±0.12
Scanner 3	4.11±0.12	3.63±0.19	1.17±0.06	3.63±0.19	1.08±0.05	3.29±0.17	1.04±0.05
Scanner 4	3.64±0.31	2.10±0.07	0.68±0.00	3.24±0.01	0.96±0.00	NA	NA
Scanner 5	3.52±1.12	2.82±0.15	0.91±0.05	2.93±0.46	0.87±0.13	2.98±0.02	0.94±0.00

Expected SUVs are measured from the dose calibrator and decay corrected. Measured SUVs are the mean SUV value extracted from PMOD. The "Average" SUV value per scanner is the averaged of the mean SUVs per site for *n* = 3 measurements, expressed as mean ± standard deviation. SUV data are also presented as normalized to the mean SUV measurement per scanner.

NA = not available; FBP = filtered back projection. ANOVA: Default *P* < 0.001, Standard *P* < 0.205, FBP *P* < 0.388 (FBP scanner 4 not included), *n* = 3 per group.

#### PET Rod

Visual and horizontal profile analysis of the collected PET rod phantom data are shown in Supplemental Figure 4. Images reconstructed with the sites’ default reconstruction methods showed that the highest measured PET image resolution was 1.2 mm, as measured in scanner 3 and 5 (Supplemental Fig. 4A). When scanner 4 and 5 PET data were reconstructed using the standardized method, 2.0 and 1.5 mm rods became well resolved in scanner 4, while scanner 5's spatial resolution remained essentially unchanged (Supplemental Fig. 4B).

### Analysis of CT Acquisitions Using Default and Standardized Protocols

#### CT Air/Water

The HU extracted using CT default acquisition protocols for scanners 2–4 were within a global average range for air of −989 ± 13 HU (mean ± SD, *n* = 3) and water 38 ± 61 HU (mean ± SD, *n* = 3). The greatest extracted HU for water was 133 and for air was −967, measured in scanner 1.

When the standardized CT protocols were applied, results for scanner 1 improved (water HU improved from 133 to −77HU), while HU water results for scanners 2–4 were all within ±30 HU from 0 HU (Table 4). The greatest measured HU for air when using CT standardized protocols was −990 HU.

**TABLE 4:** Hounsfield Units (HU) Measured Using the CT Air/Water Phantom and Default/Standardized Protocols

Scanner	Average HU water (0)	Water STDEV	Average HU air (**−**1,000)	Air STDEV
1: Default	133.05±5.94	284.35±4.07	−967.86±5.35	149.97±0.73
Standardized	−77.91±1.15	122.32±35.89	−990.46±2.72	82.21±19.61
2: Default	−29.62±0.49	32.28±0.08	−993.54±0.08	11.34±11.30
Standardized	−27.88±0.40	35.59±0.10	−993.29±0.05	12.06±0.13
3: Default	16.97±3.68	43.18±0.10	−994.98±0.61	15.76±6.10
Standardized	28.78±2.33	45.95±0.081	−996.92±0.08	7.15±0.10
4: Default	24.85±6.77	24.42±1.05	−1000±0.00	8.85E−12
Scanner 5, not calibrated to measure HU (output in linear gray scale), converted to HU				
5: Default	−10.12	64.9	−1008.26	92.03
Standardized	−3.42	142.52	−1024.19	73.47

#### CT TEM

**FIGURE 2.**
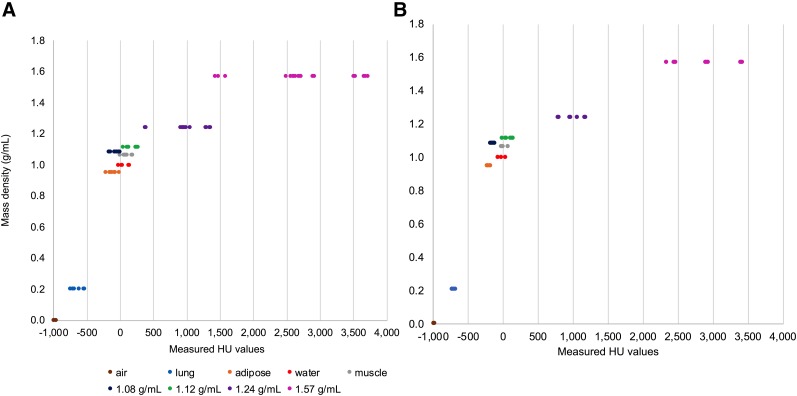
CT TEM, air and water HU results. For each material, each data point represents a measurement from a scanner (*n* = 3) from 4 different sites. Densities 1.08 to 1.57 g/mL include rod sizes 2 mm and 4 mm as reported by the manufacturer. The *x*-axis clearly shows the spread of HU values per density (A) and displays the significant variations measured using the default protocols (*P* < 0.0001, 1-way ANOVA, *n* = 3 per group). (B) Improved precision across scanners and densities when the standardized protocol is used.

A 1-way ANOVA revealed significant differences across all tissue groups (*P* < 0.0001, *n* = 3), with the greatest variability (1581 HU, i.e., scanner means ranging between 3,599 and 2,018 HU) measured in the 1.57g/mL rod when CT default methods were used to collect imaging data (Fig. 2A). The HU values measured for the 1.08 g/mL TEM rod had a mean percentage difference of 90% when default CT protocols were used, while the greatest mean percentage difference in the adipose rod was 147% between scanner 1 and 2. Two scanners showed the highest discrepancy in the HU comparison between the 4 mm and 2 mm rods of the same TEM (1.08 g/mL and 1.12 g/mL hydroxyapatite). Scanner 1 calculated percentage difference between the 4 and 2 mm 1.08 g/mL hydroxyapatite rods was 130% and scanner 3 measured percentage difference between the 4 and 2 mm 1.12 g/mL hydroxyapatite rods was 158%.

The use of a CT standardized protocol improved quantitative precision for all the materials (Fig. 2B). The greatest improvement was measured in the rods with densities of 0.21, 0.95, and 1.08 g/mL representing lung, adipose, and soft tissue, respectively. For example, the quantitative precision for the rod representing adipose tissue (0.95 g/mL) improved from a standard deviation of 77% with a COV of 66% to a standard deviation of 22% and a COV of 3% relative to the global mean. Furthermore, the lung rod measured a reduction of mean differences, in which scanner 3 improved from a mean of −728.4 HU, standard deviation of 35.16%, to a mean of −738.4 HU with a standard deviation of 0.64%. Also, the 1.57 g/mL hydroxyapatite rod's measured mean difference was reduced by 67% between scanners from 1,581 to 518 HU. The large percentage difference seen in scanner 1 between 4 and 2 mm 1.08 g/mL rods when using default protocols reduced by 109% when standardized protocols were used. However, in scanner 3, the measured percentage difference between the 4 and 2 mm 1.12 g/mL hydroxyapatite rod was essentially unchanged.

#### CT Bar

Scanners 1, 2, and 4 were unable to resolve 150 μm lines using default protocols or distinguish the sections of lines/dots patterns. Scanner 5 had the highest spatial resolution for a default protocol of 150 μm (Supplemental Fig. 5A). A slight improvement (scanners 1–3) or no change in measured spatial resolution was seen when using the CT standardized protocol (Supplemental Fig. 5B).

### Analysis of Measured Absorbed CT Radiation Dose Using Default and Standardized Protocols

#### CT Dose (CTDI)

Measured CT absorbed doses using the default protocols at each site ranged from 11 mGy to 216 mGy (Table 5). Ionizing radiation absorbed dose measurements in scanner 5 reduced by 81% when using the standard protocol. The absorbed CT doses measured was reduced by 48% (mouse phantom) and 40% (rat phantom) when using standardized CT protocols across sites.

**TABLE 5:** CT Absorbed Doses Determined Using Default Protocols and a Standardized Protocol for Mice and Rats

	Default (mGy)		Standard (mGy)		Measured dose difference default to standard (%)	
Scanner	Mouse	Rat	Mouse	Rat	Mouse	Rat
1	11±0.10	7±0.10	20±0.09	13±0.16	+77	+86
2	40±0.11	28±0.02	31±0.23	21±0.08	−23	−23
3	59±0.03	48±0.11	39±0.23	28±0.08	−34	−42
4	32±0.18	15±0.10	56±0.76	25±0.05	+71	+60
5	216±0.02	100±0.17	41±0.02	31±0.03	−81	−69

Results expressed as mean ± standard deviation, *n* = 3.

## DISCUSSION

We find the significant quantitative differences across sites routinely used default protocols concerning. For example, a commonly used analysis tool both in preclinical and clinical is the extractions of SUV measurements. An impacting factor on SUV measurements are the RCs, and as shown the RCs greatly vary using different default reconstruction protocols. This is in line with previous reports on different PET reconstruction methods on image data quantification (22–24). Discordant SUV measurements are revealed not only across sites but also internally between scanner's different reconstruction methods. Notably we measured a 54% difference in SUV for scanner 4 when changing from 12 MLEM to 25 MLEM. It was the FBP method that produced the most consistent and reproducible results across all scanners.

The literature spanning reconstruction methods (from FBP to iterative) is vast. Unfortunately, currently there is not a single solution that adequately fits all scanners due to differences in scanner manufacturing. The recently published paper by Mannheim et al. (2019) measured PET uniformity, RC, and SOR in the Siemens Inveon and Focus using the reconstruction method of 2D OSEM 4*16 (16). This method differs from both the various default reconstruction methods revealed and from the standardized protocol designed to suit 5 five different scanners in our study. Their study protocols in the Siemens platform produced similar uniformity and SOR but different RC values from the 5 scanners (reconstruction methods) in our study (16). This then begs the question of setting FBP as the standard for quantitative measurements given the improved precision of RCs and SUVs across sites. Nevertheless, using a combination of reconstructing with FBP and OSEM (as opposed to MLEM) serves the dual purpose of providing more accurate and precise quantitative information. The combined approach will also retain suitable image quality for better delineation of small organs and structures in preclinical animal species (25,26). Therefore, we recommend VOIs are drawn on the reconstructed OSEM image for better location/orientation then applied on the FBP image for accurate quantification. Based on our results, it is recommended that the total number of updates (iterations*subsets or iterations) are no less than 24 and no more than 36 for analysis of image data in conjunction with using FBP (Table 6).

**TABLE 6:** Proposed Preclinical Standard Protocols for Daily Routine Use Irrespective of Scanner/Site

Parameter	Protocol
PET reconstruction	Iterative algorithms OSEM or MLEM total updates (iterations*subsets or iterations) to be in the range of 24 to 36. FBP is also recommended for use in conjunction with iterative methods.
CT image acquisition parameters for FBP reconstruction methods	Tube voltage at 50 kVp Number of projections at 360 Exposer of 300 ms.

Unlike PET, the CT image reconstructions were all done, default and standardized, with the FBP method. Though like PET, the quantitative biases revealed with the default protocols were substantial. The significant variations in HU from the various CT default protocols reiterates the necessity of standardization. In this case the CT acquisition protocols have a more prominent role than reconstruction methods. Applying a standard CT acquisition protocol improved quantification precision of HU values across sites for each TEM measured as well as in air and water. The recommended standard CT protocol sets the tube voltage at 50 kVp for 300 ms with 360 projections (Table 6). This recommendation is completely feasible given that every scanner enrolled in this study is capable of those parameters. However, it is important to emphasize the need for scanner calibration. Initially more than one scanner was plagued by calibration errors requiring intervention from the scanner manufacturer. Therefore, along with setting a CT protocol, correct calibration (HU values) at the different tube voltages needs to be ensured.

Furthermore, not until this study has the range of HUs values been measured at preclinical CT voltages. The traditional HU scale was established using clinical protocols with a higher tube voltage than 50 kVp (27). The average HU values we report here per TEM across multiple scanners can be used to establish preclinical HU ranges (Supplemental Table 5).

Our CT absorbed dose results indicate standardized protocols produce a reduction of the average absorbed ionized radiation received by small laboratory animals, with no image degradation. Unfortunately, the change in tube voltage to 50 kVp in scanner 1 from 40 kVp and scanner 4 from 35 kVp with increased projections (250 to 360) led to an increase in the absorbed radiation dose. The amounts measured in the mouse and rat were increased by 77% and 86% in scanner 1. Scanner 4 measured an increase of 71% and 60% in the mouse and rat, respectively. However, even with the increase in scanner 1 and 4 all measured absorbed doses are now under limits of damaging ionizing radiation absorbed doses reported in the literature (<60 mGy) (13,18,28,29). Critically, the measurements reported here provide a foundation for regulations regarding CT absorbed radiation doses. It should be noted that in the clinical setting absorbed radiation doses have been regulated since the 1950s (30). Implementing radiation dose regulations preclinically will therefore reduce cumulative severity and animal suffering while reducing the potential impact radiation may have on results, especially in longitudinal studies.

## CONCLUSION

Empirical PET and CT quantitative data variability reduces when standardized protocols are used. Adopting the suggested standardized protocol establishes continuity, allowing for diagnostic and therapeutic agents to be developed and tested across imaging platforms with consistency. Data showed that standardization improves precision and accuracy in CT image quantification, while reducing the impact of absorbed ionizing radiation dose to small laboratory animals. Standardization will provide more robust, reliable, and reproducible translational preclinical PET/CT imaging data sets. Therefore, this phantom work provides the foundational mainframe towards improving reproducibility of in vivo PET/CT measurements irrespective of scanner manufacturer.

## DISCLOSURE

We thank the National Centre for the Replacement, Refinement and Reduction of Animals in Research (NC3Rs) for funding this work (Studentship grant NC/P00170X/1) to Wendy McDougald. Adriana Alexandre S. Tavares is funded by the British Heart Foundation (RG/16/10/32375). The British Heart Foundation is greatly acknowledged for providing funding toward establishment of the Edinburgh Preclinical PET/CT laboratory (RE/13/3/30183). No other potential conflict of interest relevant to this article was reported.

**KEY POINTS**

**QUESTION:** Will standardization of preclinical PET/CT protocols across multiple scanners reduce quantitative bias in image data while maintaining image quality?
**PERTINENT FINDINGS:** When each scanner’s default protocols were used, results unequivocally showed substantial and significant quantification bias across all scanners for all CT and PET outcome measurements, including image quantification, resolution, uniformity, spillover ratios, and absorbed dose. Developed and tested standardized preclinical PET/CT protocols improved accuracy and precision on all evaluations.
**IMPLICATIONS FOR PATIENT CARE:** Implementing preclinical PET/CT standards produces more reliable and robust translational datasets, ultimately improving the success of clinical studies and applications.
